# Deletion of Kvβ2 (AKR6) Attenuates Isoproterenol Induced Cardiac Injury with Links to Solute Carrier Transporter SLC41a3 and Circadian Clock Genes

**DOI:** 10.3390/metabo11040201

**Published:** 2021-03-29

**Authors:** Jared Tur, Kalyan C. Chapalamadagu, Ravikumar Manickam, Feng Cheng, Srinivas M. Tipparaju

**Affiliations:** Department of Pharmaceutical Sciences, Taneja College of Pharmacy, University of South Florida, Tampa, FL 33612, USA; jaredturphd@gmail.com (J.T.); ckchapala@gmail.com (K.C.C.); ravikumarm@usf.edu (R.M.); fcheng1@usf.edu (F.C.)

**Keywords:** Kvβ subunit, redox, potassium channel, heart, aldo-keto reductase, pyridine nucleotides, action potential, magnesium

## Abstract

Kvβ subunits belong to the aldo-keto reductase superfamily, which plays a significant role in ion channel regulation and modulates the physiological responses. However, the role of Kvβ2 in cardiac pathophysiology was not studied, and therefore, in the present study, we hypothesized that Kvβ2 plays a significant role in cardiovascular pathophysiology by modulating the cardiac excitability and gene responses. We utilized an isoproterenol-infused mouse model to investigate the role of Kvβ2 and the cardiac function, biochemical changes, and molecular responses. The deletion of Kvβ2 attenuated the QTc (corrected QT interval) prolongation at the electrocardiographic (ECG) level after a 14-day isoproterenol infusion, whereas the QTc was significantly prolonged in the littermate wildtype group. Monophasic action potentials verified the ECG changes, suggesting that cardiac changes and responses due to isoproterenol infusion are mediated similarly at both the in vivo and ex vivo levels. Moreover, the echocardiographic function showed no further decrease in the ejection fraction in the isoproterenol-stimulated Kvβ2 knockout (KO) group, whereas the wildtype mice showed significantly decreased function. These experiments revealed that Kvβ2 plays a significant role in cardiovascular pathophysiology. Furthermore, the present study revealed SLC41a3, a major solute carrier transporter affected with a significantly decreased expression in KO vs. wildtype hearts. The electrical function showed that the decreased expression of SLC41a3 in Kvβ2 KO hearts led to decreased Mg^2+^ responses, whereas, in the wildtype hearts, Mg^2+^ caused action potential duration (APD) shortening. Based on the in vivo, ex vivo, and molecular evaluations, we identified that the deletion of Kvβ2 altered the cardiac pathophysiology mediated by SLC41a3 and altered the NAD (nicotinamide adenine dinucleotide)-dependent gene responses.

## 1. Introduction

The Kvβ subunits belong to the aldo-keto reductase superfamily and were previously demonstrated to bind pyridine nucleotides and modulate the cardiac function [1,2]. Previous studies have reported the multifunctional binding of Kvβ subunits with cortisones, as well as other protein targets such as MHC7 using in vitro, heterologous cell models and in vivo investigation [3,4]. Genetic mutation studies in humans and mice associate Kvβ2 to various neurological disorders, including epilepsy [5,6]. In the neuronal system, the deletion of Kvβ2 leads to associative memory impairment and amygdala hyperexcitability. Perkowski et al. presented that 1p36 deletion syndrome and Kvβ2 deletion exhibit similarities in terms of the cognitive and neurological impairments observed in the patients with this disease [5,7]. Recently, our group demonstrated the significant cardiac alterations observed in Kvβ2 knockout (KO) mice. Kvβ2 KO mice presented with decreased repolarization reserves manifested in increased QTc durations coupled with elevated action potential durations [8]. 

Kvβ subunits (Kvβ1-3) are accessory subunits that bind to and modulate the Kv channel gating and kinetics. Recent reports have identified that Kvβ subunits play a larger role and exhibit binding to multiple binding partners and, therefore, may offer key physiological roles within the heart. To date, multiple binding partners have been identified to associate with the Kvβ2 subunit, including numerous Kv channels, MHC7, pyridine nucleotides, and TRPV1 [9,10,11]. However, the pathophysiological relevance of Kvβ2 in view of cardiac hypertrophy or acute insult, such as ischemia-reperfusion, remain largely unknown. In addition to modulating Kv channels, Kvβ2 subunits also bind pyridine nucleotides (NAD[P]^+^/NAD[P]H) with high affinity [8]. Heterologous expression studies have shown that Kvβ subunits differentially regulate Kv channels, depending on the oxidative state of the bound pyridine nucleotide molecules [12,13,14]. While the reduced pyridine nucleotides (NAD(P)H) sustain or accelerate the Kvβ inactivation of Kv currents, the oxidized nucleotides [NA(D)P^+^] abolish inactivation, and gating [2]. Thus, it has been suggested that Kvβ subunits act as a biochemical link between the Kv channel function and cellular metabolic status. Previous studies showed that pyridine nucleotides (NAD(H)/NAD(P)H) serve as intracellular mediators of electron transfer and regulators of substrate flux, mitochondrial respiration, cell survival, ion channel homeostasis, and oxidative stress [2,15,16]. Altered levels of reduced and/or oxidized forms of pyridine nucleotides result in cellular redox stress and cardiac pathology. Pressure- or agonist-induced cardiac hypertrophy in mice showed that the cardiac pathophysiologies involve significant changes in the redox status of pyridine nucleotides. Studies have shown that cardiac NADH/NAD (nicotinamide adenine dinucleotide reduced/nicotinamide adenine dinucleotide oxidized) ratio significantly increases in ischemia-reperfusion injuries [17]. In cases of NAD depletion, the exogenous supplementation of NAD reverses the agonist-induced hypertrophy in the heart or in cultured neonatal myocytes [17,18]. Studies in isolated hearts also show that ischemia alone increases NADH levels [19]. As pyridine nucleotides appear to be a frequent denominator in the pathogenesis of heart diseases, understanding the cellular mechanisms involving redox imbalance can help mitigate the role of redox stress in cardiac pathophysiologies, which is of clinical and pharmaceutical significance. Kvβ subunits may serve as a crucial link in interventions aimed at understanding and/or mitigating cardiac pathophysiologies.

Microarray and qPCR analyses have revealed novel molecular targets that may play a major role in how Kvβ2-deleted mouse hearts respond to pathophysiological stimuli. In terms of the redox and sensing of NADH/NAD changes, we previously demonstrated that the lack of Kvβ1 subunit is sufficient to modulate the electrical changes in the heart. However, the role of Kvβ2 under such pathological scenarios remains unknown [10]. Therefore, in the present study, we investigated the pathological roles of Kvβ2 using isoproterenol-infused cardiac hypertrophy. Cardiac function was evaluated with echocardiography and electrocardiography, and the action potentials were measured to identify the changes associated with deletion of the Kvβ2 subunit in cardiac pathophysiology in the mouse and whether the genes responsible for such alterations play a role in cardiac responses.

## 2. Results

### 2.1. Echocardiographic Assessment Demonstrates a Differential Change in Cardiac Injury in Kvβ2 KO Mice by Isoproterenol Infusion

**Figure 1:**
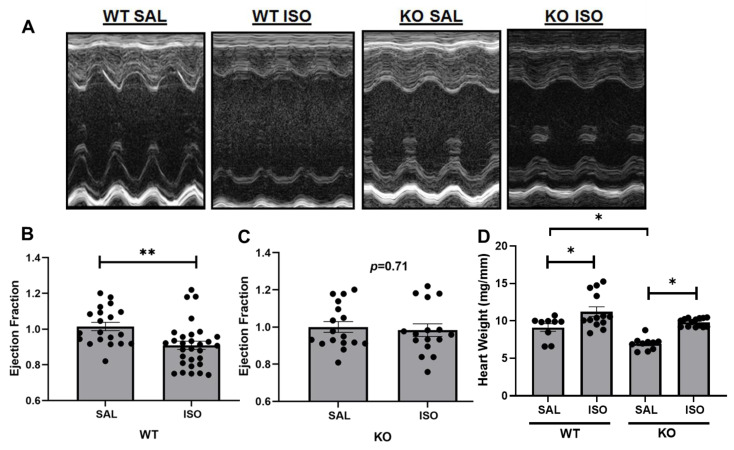
Isoproterenol-induced cardiac hypertrophy and functional alterations. Echocardiography changes in wildtype (WT) and Kvβ2 knockout (KO) saline (SAL) and isoproterenol (ISO)-treated mice. (**A**) Representative M mode images of the left ventricular chamber. (**B**) Ejection fraction (EF) determined by the M mode measurements in wildtype mice. The EF values are normalized to wildtype saline. (**C**) Ejection fraction (EF) determined by M mode measurements in knockout mice. The EF values are normalized to knockout saline. (**D**) Heart weight measurements normalized with the tibia length. The data represented are the mean ± SEM. ** represents, *p* < 0.01, * represents *p* < 0.05.

WT mice exposed to two weeks of treatment of isoproterenol resulted in a significant decrease in the ejection fraction (EF%) compared with saline-infused WT mice (Figure 1A,B). Kvβ2 KO mice demonstrated no significant decreases in EF% after two weeks of treatment of isoproterenol compared with saline-infused KO mice (Figure 1A,C). In line with our previously published data, Kvβ2 KO mice presented with smaller hearts, as evident by the significant decrease in heart weight (Figure 1D). Heart weights in both the Kvβ2 KO mice and wildtype mice demonstrated a significant increase in heart weight after isoproterenol exposure with a clear hypertrophic response (Figure 1D).

### 2.2. Kvβ2 Deletion Results in Significant Prolongation in QTc While Attenuated during Isoproterenol-Induced Cardiac Injury

**Figure 2:**
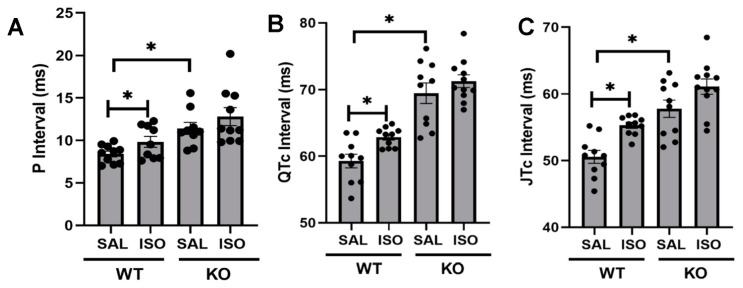
Isoproterenol-induced electrical prolongation ablated in Kvβ2 KO mice. (**A**) P interval. (**B**) QTc interval. **(C**) JTc interval. The data represented are the mean ± SEM. * represents *p* < 0.05.

In confirmation with our previously published work [8], Kvβ2 KO mice aged 14–16 weeks old demonstrated significantly prolonged P intervals (Figure 2A) compared with the wildtype controls. Isoproterenol exposure did not demonstrate a significant effect on the P interval either in wildtype or Kvβ2 KO mice. The QTc intervals demonstrated significant prolongation compared with the WT control (saline) mice (Figure 2B). The JTc (corrected JT) interval was also calculated as a measure of the ventricular repolarization, demonstrating significant prolongation compared with WT control (saline) mice (Figure 2C). Two weeks of treatment of isoproterenol resulted in a significant prolongation in the QTc and JTc intervals in WT mice (Figure 2B,C). Interestingly, two weeks of treatment of isoproterenol in KO mice demonstrated no further prolongation in the QTc or JTc intervals.

### 2.3. Changes in Monophasic Action Potentials in ISO-Infused Hearts

**Figure 3:**
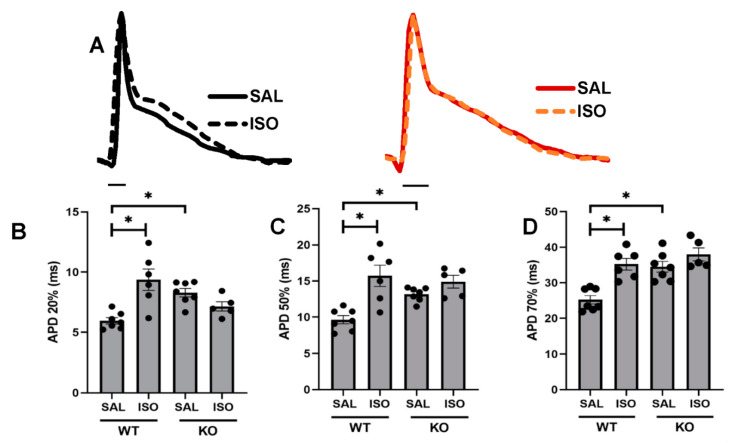
Isoproterenol-induced monophasic action potential (MAP) prolongation was ablated in Kvβ2 KO mice. (**A**) Representative MAP traces from WT (black lines) and Kvβ2 KO (red lines) hearts (14-16 weeks of age). Solid line indicates saline (SAL) treatment, while dotted line indicates isoproterenol (ISO) treatment for 14 days; scale bar represents 10 milliseconds. (**B**) Left ventricular (LV) surface MAPs at 20% repolarization (APD 20%). (**C**) LV surface MAPs at 50% repolarization (APD 50%). (**D**) LV surface MAPs at 70% repolarization (APD 70%). The data represented are the mean ± SEM. * represents *p* < 0.05.

The monophasic action potential (MAP) recordings demonstrated a significant prolongation in the action potential durations (APDs) in Kvβ2 KO mice compared with the WT controls (saline) (Figure 3A–D). The wildtype hearts that were exposed to two weeks of treatment of isoproterenol resulted in a significant prolongation in the APD values, including 20%, 50%, and 70% repolarization (Figure 3B–D). The Kvβ2 KO hearts, however, demonstrated no further prolongation in the APD values at 20–70% repolarization compared with Kvβ2 KO (saline).

### 2.4. Kvβ2 Deletion Results in Significant Induction of Ventricular Tachycardias and Increased APDs

**Figure 4:**
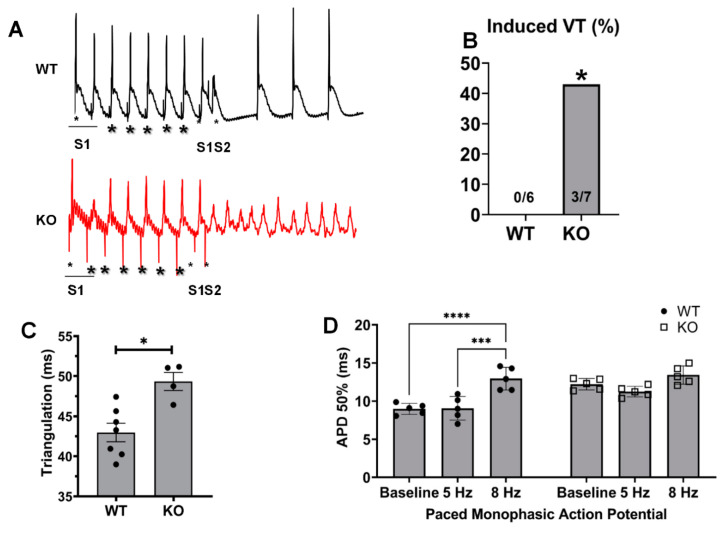
Kvβ2 deletion increases the susceptibility to ventricular arrhythmias. (**A**,**B**) Programmed electrical stimulation in wildtype (WT) and Kvβ2 knockout (KO) mice. Onset of ventricular tachycardia (VT) after premature stimulus (small asterisk) is shown in Kvβ2 KO mice (none of the six wildtype mice were inducible, but three of the seven KO mice were inducible: * *p* < 0.05). (**C**). LV surface MAP triangulation at the baseline measurements. (**D**) LV surface MAPs at 50% repolarization (APD 50%) during paced stimulations at 5 Hz and 8 Hz. Scale bar represents 100 ms. The data represented are the mean ± SEM. * represents *p* < 0.05, *** represents *p* < 0.01, and **** represents *p* < 0.001.

We examined whether excessive prolongation of the repolarization could alter the arrythmia susceptibility in Kvβ2 KO mice. The use of external programmed electrical stimulation resulted in a marked increase in the occurrence of ventricular arrhythmias seen in Kvβ2 KO hearts (Figure 4 A,B). The baseline monophasic action potential recordings demonstrated a significant increase in the triangulation duration in Kvβ2 KO hearts compared with the wildtype control (Figure 4C). Further, the paced monophasic action potential recordings demonstrated a significant increase in APD 50% in WT mice at 8 Hz (Figure 4C), while Kvβ2 KO mice demonstrated no significant differences in APD 50% at 5 or 8 Hz (Figure 4C), thus demonstrating that the genetic ablation of Kvβ2 leads to baseline action potential prolongation compared with the WT hearts; however, upon stimulation, the Kvβ2 KO mouse hearts lacked the ability to increase the action potential duration, demonstrating that Kvβ2 may play a role in the excitability of the heart. In addition, the deletion of Kvβ2 leads to an increased propensity of developing induced ventricular tachycardia.

### 2.5. Microarray Analysis Identifies Novel Genes Altered in Kvβ2 KO Mice

**Figure 5:**
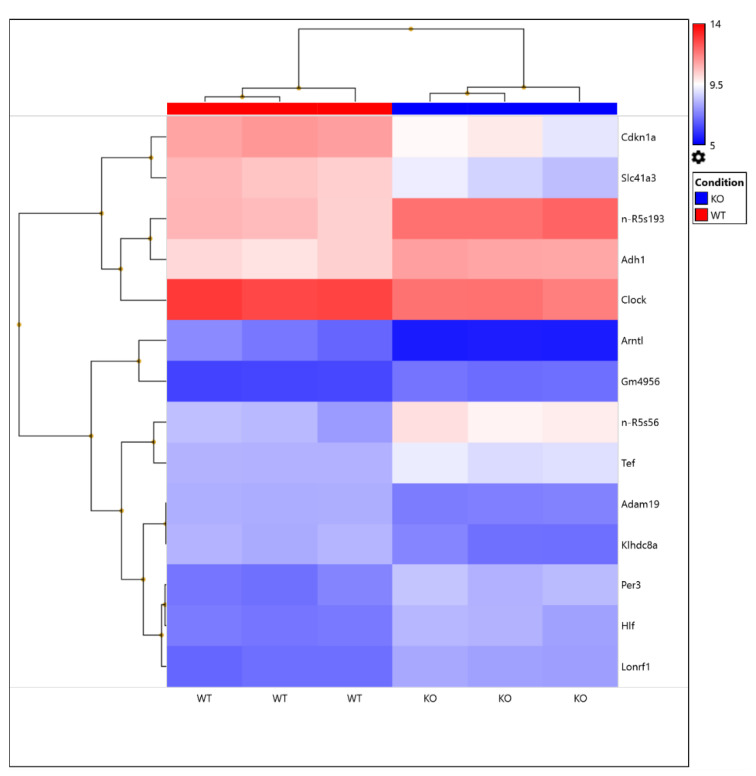
Heatmap of the differentially expressed genes identified (log2-transformed). Dendrograms were also added to the rows (genes) and columns (samples) to show the clustering results. The color palette “blue–red” was used.

**Figure 6:**
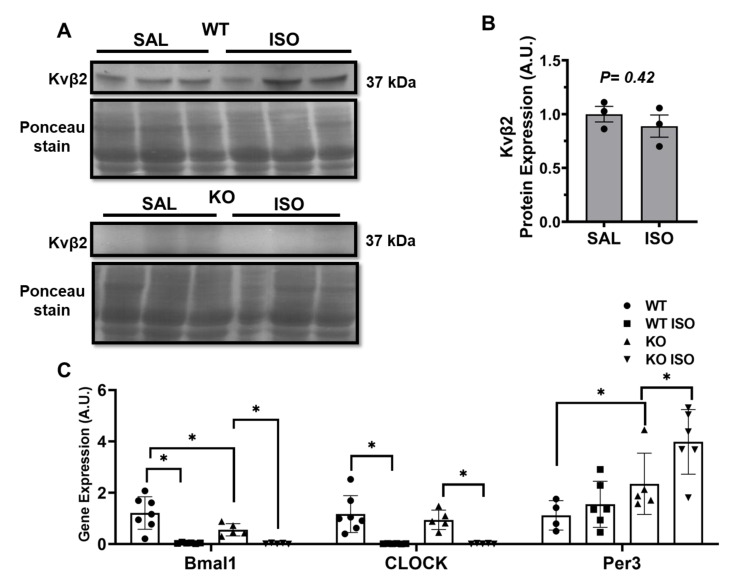
Circadian gene alterations induced in Kvβ2 (voltage gated potassium channel subunit β2) deletion. (**A**) Protein expression of Kvβ2 in wild type (WT) and knockout (KO) hearts treated with saline (SAL) and isoproterenol (ISO). (**B**) Quantitative Kvβ2 protein expression from wildtype (WT) hearts, (KO hearts demonstrated no measurable protein expression for Kvβ2). The data represented are the mean ± SEM. (**C**) PCR (polymerase chain reaction) expression of the key circadian genes selected from the microarray. The data represented the mean ± SEM. * represents *p* ≤ 0.05.

The transcriptome analysis of cardiac samples from WT and KO mice demonstrated significant alterations in 156 gene expressions (Figure 5), allowing the identification of 138 upregulated genes and 18 downregulated genes that were utilized for further analysis. The top genes altered are presented in Figure 5, based on the statistical analysis using a cutoff of 1.5-fold or higher and *p*<0.05 for comparing WT vs KO. The transcriptome analysis identified a novel solute carrier transport protein SLC41a3 as a likely downregulated gene that was modulated in the Kvβ2 KO mouse hearts. In addition, the transcriptome analysis revealed significant alterations in the peripheral cardiac circadian core clock genes (Figure 5). The examination of the Kvβ2 expression in the presence of isoproterenol-induced injury resulted in no significant expression changes (Figure 6A,B). We, therefore, confirmed the key transcriptome targets with qPCR analysis and identified that Arntl (Bmal1; Brain and Muscle ARNT-Like 1) and Clock (Circadian Locomotor Output Cycles Kaput) were downregulated in Kvβ2 KO hearts, while Per3 (Period Circadian Regulator 3) was significantly upregulated (Figure 6C). A further examination investigating isoproterenol’s role resulted in a significant decrease in Arntl/Baml1 and Clock gene expressions in both WT and Kvβ2 KO hearts, while isoproterenol’s effect resulted in a marked increase in the Per3 expression levels (Figure 6C). Overall, the microarray and qPCR validation identified the gene responses that were altered due to Kvβ2 KO and were further modulated due to isoproterenol exposure.

### 2.6. Kvβ2 KO Results in Significant Decrease in SLC41a3 Expression

**Figure 7:**
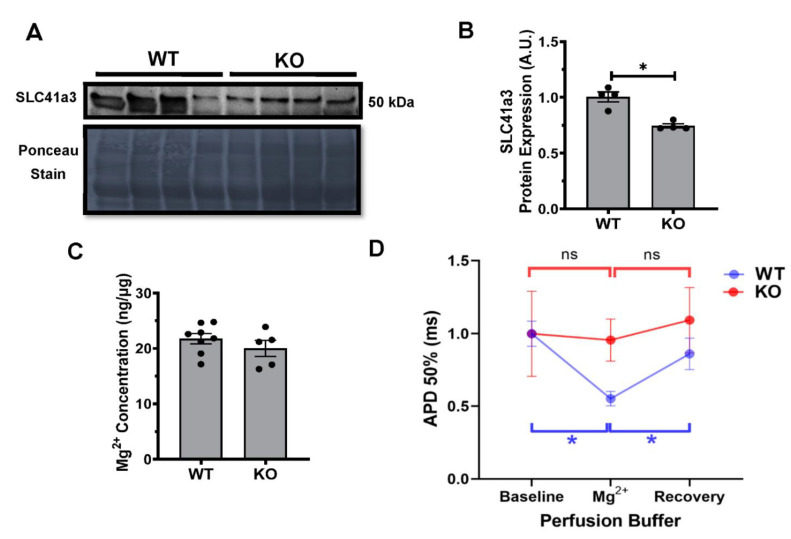
SLC41 expression and Mg^2+^ concentration alters the monophasic action potentials (MAPs). (**A**) Protein expression of SLC41a3 in WT and KO hearts. (**B**) Quantitative SLC41a3 protein expression from WT and KO hearts are the mean ± SEM. * represents *p*≤0.05. (**C**) Serum magnesium concentrations from WT and KO mice are the mean ± SEM. (**D**) LV surface MAPs at 50% repolarization (APD 50%) in WT hearts (blue) and KO hearts (red) normalized to baseline measurements, followed by exposure to MgSO_4_ (Mg^2+^) following a recovery period with the original buffer. The data represented are the mean ± SD. * represents *p* < 0.05.

The protein analysis from the WT and KO hearts confirmed a significant decrease in the expression levels of SLC41a3 (Figure 7A,B). The serum samples collected from WT and KO mice demonstrated no significant differences in the Mg^2+^ concentration (Figure 7C). To assess the potential electrical alterations associated with the decreased SLC41a3 expression, we performed monophasic action potential recordings in the presence and absence of elevated Mg^2+^ utilizing MgSO_4_. In WT hearts, 3-mM Mg^2+^ exposure demonstrated a significant decrease in the APD 50% duration when compared with the baseline measurements. The subsequent washout of the 3-mM Mg^2+^ and recovery phase returned the APD 50% values to those seen during the baseline (Figure 7D). The KO hearts exposed to similar experimental conditions demonstrated no significant differences during the 3-mM Mg^2+^ exposure, as well as no recovery phase return (Figure 7D).

## 3. Discussion

In the present study, we identified that the genetic absence of Kvβ2 prolonged cardiac repolarization induced during cardiac hypertrophy by isoproterenol infusion are preserved from further prolongation, thus suggesting a key role for Kvβ2 in cardiac pathophysiology and repolarization. Kvβ2 deletion resulted in significantly altered cardiac electrical parameters, including the QTc interval and left ventricular action potential durations. Although the deletion of Kvβ2 itself leads to cardiac electrical changes, under isoproterenol-infused conditions, we identified no further exacerbations in the cardiac electrical alterations when compared with the wildtype controls. Previous studies from our laboratory investigate the Kvβ2 subunit responses to redox alterations [10]. Therefore, in the present study, we sought to investigate if there were members in the redox system playing a role in the preserved electrical activity in Kvβ2 knockout mice.

In the cardiovascular system, the significance of Kvβ2 remains relatively unknown; previously, we reported that, at the physiological level, the Kvβ2 subunit senses the changes in pyridine nucleotides and modulates the ion channel activity [8]. In addition, reports have also provided an important role for Kvβ2 in the brain and pulmonary system [5,20]. Subsequent studies in mice have confirmed that Kvβ2 deletion leads to neurological hyperexcitability, supporting that Kvβ2 is essential for K^+^ conduction and cellular repolarization [5]. Given the abundance of evidence of Kvβ2 binding and modulating Kv channel gating and activity [11,21], our data suggests an essential role of Kvβ2 in cardiac phenotype determination and potential pathophysiological responses. Heterologous expression studies and our recent study have demonstrated that Kvβ2 binds to Kv channels and modulates the electrical changes in the heart [8]. These interactions may be the primary reason why Kvβ2 deletion abolishes pyridine nucleotide sensing in isoproterenol-infused mouse hearts. Cardiac excitability is regulated by ion channels and β-subunits; we demonstrated that a lack of Kvβ2 abolished the frequency-dependent increase in the action potential duration and an overall higher propensity of KO hearts to induced ventricular tachycardia. Therefore, the present study highlighted that Kvβ2 plays an important role in cardiac excitability, and the deletion of Kvβ2 leads to prolonged QTc and an increased susceptibility to cardiac arrhythmias. Further examination of the repolarization phase in Kvβ2 KO mice demonstrates a significant prolongation in JTc intervals. The reports highlight that the JTc interval has been utilized as a more accurate depiction of the repolarization phase, because it subtracts the QRS duration and, therefore, the depolarization phase taken into account in the QTc measures [22]. A closer examination of Kvβ2 KO monophasic action potential recordings demonstrates a significant increase in the triangulation durations compared with the wildtype controls. Triangulation has been linked as a reflection of the action potential shape with an increasing duration demonstrating a greater prolongation in the repolarization phase of the cardiac action potential [23] and a potential decrease in the total ERP (effective refractory period), thus lending itself to a proarrhythmic state and potential EAD (early after depolarization) [24]. Earlier reports suggest that a strong link between APD prolongation and triangulation or instability are proarrhythmic, while just APD prolongation alone may be antiarrhythmic [25]. While previous investigations have highlighted Kvβ2 deletion leading towards Kv channel alterations, we sought additional global examination through a microarray. The gene array analysis for identifying the molecular changes due to Kvβ2 deletion pointed to two key areas that may be directly modulated: (A) NAD-dependent genes, including the heart circadian Clock, Bmal1 and Per3, and (B) a downregulation of the Carrier or solute transporter SLC41a3 in Kvβ2 knockout hearts. The increase in Per3 could negatively regulate Bmal1 and Clock through the negative feedback mechanism [26]. These observations lend to the idea that Kvβ2 may be responsible for additional ways beyond pyridine nucleotide sensing and protein–protein interactions for myriad effects at the gene level [27]. The NAD-dependent gene alterations in the Kvβ2 knockout mice identified that the Kvβ2 (AKR6) gene is tightly regulated by the pyridine nucleotide gene upstream of its protein–protein functions. The identification of key genes that are modulated in Kvβ2-deleted mice provide new insights into the modulation of Bmal1, Clock, and Per3 expression in the heart and might be of significance in the peripheral cardiac circadian rhythm, the alterations of which could lead to cardiac pathophysiology associated with cardiovascular diseases.

In the heart, the role of SLC41a3 is largely unknown; however, the protective role of related members from the SLC41 [28] superfamily was demonstrated to inhibit angiotensin II-induced cardiac fibrosis via decreased magnesium efflux process and calcium signaling. The authors proposed the idea that the extrusion of magnesium via SLC41a1 may be involved in alleviating angiotensin II-induced fibrosis, since silencing SLC41a1 decreased the cardiac fibrosis induced by angiotensin II [29]. A recent investigation identified a unique functional interaction between the Kv channels, Kvβ, and SLC7a5, a neutral amino acid transporter. Lamothe et al. demonstrated a profound alteration in the expression and function of Kv1.2 when co-expressed with Kvβ and SLC7a5 [30]. In the present study, we identified that the deletion of Kvβ2 led to a decreased expression of SLC41a3, which is a distinct member of the magnesium solute carrier transport family (SLC41) and, therefore, further evaluated the magnesium effects in the Kvβ2 knockout hearts. An early investigation into the exogenous cardiac exposure to magnesium resulted in distinct and robust phenotypes. The clinical investigation demonstrated magnesium sulfate given during acute ischemia and, as an antiarrhythmic, resulted in a significant decrease in the action potential duration [31,32]. The preclinical treatment of isolated myocytes demonstrated a robust and concentration-dependent response with varying concentrations of magnesium sulfate (3–10 mM), resulting in an initial APD prolongation switching to APD reduction with the increasing concentrations of magnesium sulfate [33]. The perfusion of magnesium sulfate resulted in a significant decrease in the action potential duration in the wildtype hearts, while the cardiac action potentials were unchanged in the Kvβ2 KO hearts, suggesting that the Kv–Kvβ2–SLC41a3 axis was likely driving the action potential changes. Based on our results, along with the literature evidence, we identified that the deletion of Kvβ2 results in a decreased SLC41a3 expression, which may be responsible for further preventing the isoproterenol-induced pathological effects in the heart. However, additional investigations are required to precisely establish the molecular identify and mechanism leading to Kvβ2 mediated changes in the heart.

Further, the consistent observation of differential repolarization changes in WT and KO mice with isoproterenol-exposed conditions suggests that Kvβ2 is an important mediator of cardiac repolarization under chronic stress with an isoproterenol infusion that alters the redox status of pyridine nucleotides [10], the responses of which are mediated—at least, in part—by SLC41a3. Hence, the current study offers novel gene responses and insights into the role of the Kvβ2 subunit in the mediation and regulation of cardiac pathophysiology.

## 4. Material and Methods

### 4.1. Animals

Kvβ2 hemizygous mice were obtained from Dr. Geoffrey Murphy (University of Michigan, Ann Arbor, MN, USA) [5,22]. The Kvβ2 knockout (KO) mice and littermate wildtype (WT) mice were bred in-house. Male mice of 12-16 weeks of age were used in this study and fed with food and water ad libitum. All animal work was approved in advance by the Institutional Animal Care and Use Committee at the University of South Florida, Tampa, FL, USA.

### 4.2. Mouse Model of Cardiac Hypertrophy

Age-matched Kvβ2 KO and littermate wildtype mice were infused with either saline or isoproterenol hydrochloride (ISO) (Sigma-Aldrich, St. Louis, MO, USA) for 14 days at a dose of 30 mg/kg/day using osmotic mini-pumps (Alzet, Durect; model 2002, Cupertino, CA, USA) according to the previously published reports [10,24]. Mice were anesthetized with 2.5% isoflurane (Butler Schein, Dublin, OH, USA); pumps were placed subcutaneously and monitored for 14 days.

### 4.3. Echocardiography

Serial transaortic echocardiography was performed with isoflurane (2% to 3%). Mice were depilated and placed on a heated platform for imaging. Measurements were taken from three different cardiac cycles and averaged for each mouse. Calculations were performed as previously described [25]. Briefly, the ejection fraction was calculated as previously described [7,26].

### 4.4. Electrocardiography

Mice were anesthetized with 2% to 3% isoflurane/oxygen anesthesia, and lead–II electrocardiography (ECG) was recorded with a Power lab (AD Instruments, Sydney, Australia) amplifier and data acquisition system; analysis was performed by using Labchart 7.2. The end of the T wave was fixed at the point where the waveform returned to the isoelectric line, and the ECG parameters, including QTc, were assessed as reported before [24,25,27,28]. The JTc interval was calculated by the QTc minus QRS duration.

### 4.5. Monophasic Action Potentials

Monophasic action potentials (MAPs) were recorded from ex vivo heart preparations as reported before [25,28]. Mice were injected with 1-mg heparin (180 USP, Sigma-Aldrich, St. Louis, MO, USA) and euthanized with Somnasol (pentobarbital sodium, 50-mg/kg body weight, Henry Schein Animal Health, Dublin, OH, USA) by intraperitoneal (i.p.) injection. Hearts were isolated through a bilateral thoracotomy and retrograde perfusion with Krebs–Hanseleit buffer (mM: NaCl 119, NaHCO_3_ 25, KCl 4, KH_2_PO_4_ 1.2, MgCl_2_ 1, CaCl_2_ 1.8, D-glucose 10, and sodium pyruvate 2, pH 7.4) was carried out at a constant flow rate of 2.0 mL/min, 37 °C; the perfusion pressure was recorded by an in-line monitoring system for aortic pressure maintained at 70–90 mmHg. Monophasic action potentials were recorded from the left ventricular (LV) epicardial surface using a contact electrode (Harvard Apparatus, Holliston, MA, USA). Hearts were stabilized for 10 min, and MAP data were acquired using the 8-channel PowerLab system (AD Instruments, Sydney, Australia). Programmed electrical stimulation was performed as described previously [29,30]. Briefly, excised hearts were perfused with the Krebs–Hanseleit buffer and stabilized for 10 min; after which, the baseline MAPs were recorded. Subsequently, hearts were electrically stimulated using a platinum electrode placed on the epicardial surface, and electrical stimulation was applied using the S1-S2 protocol (Harvard Apparatus) connected with a stimulator (Powerlab, AD instruments). The S1–S2 protocol was developed by intervals initially equal to the pacing interval, and after a brief duration, the S2 cycle was progressively reduced by 1 ms to evoke the triggered arrhythmia. Adjusting the levels of the magnesium concentrations was performed with the addition of 3-mM magnesium sulfate in Krebs–Hanseleit buffer. Baseline MAPs from LV were acquired with normal buffer conditions without MgSO_4_. Subsequently, hearts were perfused for 20 min with a modified Krebs–Hanseleit buffer containing 3 mM of MgSO_4_ to acquire altered MAPs. Finally, the hearts were switched back to the perfusion of normal Krebs–Hanseleit buffer to establish the recovery signals and a return to the baseline measurement MAPs. MAPs were acquired using the 8-channel PowerLab system (AD Instruments, Sydney, Australia).

### 4.6. Microarray

Cardiac apex tissue samples from wildtype and knockout mice were utilized for analysis. RNA was extracted using a Trizol reagent and RNA easy mini kit (Qiagen, Hilden, Germany). The total RNA was amplified and labeled for the transcript analysis using an Affymetrix labeling kit (Affymetrix, Santa Clara, CA, USA). The Affymetrix Gene chip mouse array was hybridized and labeled. The signal intensity for fluorescence was captured by an Affymetrix Gene Chip Scanner.

### 4.7. Quantitative Real-Time-PCR 

Total RNA was isolated from the left ventricles of hearts using the Exiqon miRCURY RNA Isolation kit (Exiqon, Woburn, MA, USA) according to the manufacturer’s protocols. Complimentary DNA from total RNA was synthesized, and a quantitative real-time PCR (qRT-PCR) analysis was performed on the key transcriptome targets identified from the microarray, including the circadian core clock genes like Arntl (Bmal1), Clock, and Per3. The cDNA (complementary DNA) synthesis and qRT-PCR procedures were performed as described previously [25,26]. The expression of mouse Gapdh was used as an internal control.

### 4.8. Western Blots

Protein extracts from whole hearts were isolated and quantified as described previously [26,28] for the Western blot analysis. Proteins were detected with a dilution of the primary antibody as follows: 1:1000 (SLC41a3) and 1:500 (Kvβ2). Primary antibodies were obtained from SLC41a3 (Fisher Scientific, Hampton, NH, USA) and Kvβ2 from Neuromab (Davis, CA, USA). Immunoblots were quantified using ImageJ software (National Institute of Health, Besthesda, MD, USA) and mean (± SEM) values were plotted.

### 4.9. Serum Magnesium (Mg^2+^) Concentrations

Serum extracts from whole blood were isolated and quantified according to the manufacturer’s protocols using the Magnesium Assay Kit (Millipore Sigma, Darmstadt, Germany). Briefly, serum samples were added directly to a well in duplicates following the addition of Master Reaction Mix and incubation at 37 °C for 10 min. The absorbance was measured at 450 nm using a BioTek (Winooski, VT, USA) plate reader for 15 min, with readings taken every 5 min.

### 4.10. Microarray Data Analysis

The CEL files were imported into the Transcriptome Analysis Console (TAC) Software (version 4.0.2) from Affymetrix company to perform the quality control check, sample normalization, gene probe annotation, and the identification of the differentially expressed genes. All the QC (Quality Control) metrics reported no outlier samples. Default parameters and algorithms in the TAC software were used for the normalization and the statistical tests for differential expression. Genes with adjusted *p*-values< 0.05 were chosen as the significant differentially expressed genes. The heatmap of those genes was constructed in the TAC.

### 4.11. Statistical Analysis

Statistical analyses were performed with GraphPad Prism 5 (San Diego, CA, USA). A one-way ANOVA with Tukey’s multiple comparison post-hoc test was utilized for all groups. A two-way ANOVA with Tukey’s multiple comparison post-hoc test was utilized for paced monophasic action potential measurements and the qPCR analysis of circadian core clock genes expression. An unpaired *t*-test was utilized when comparing only two groups. Each dot in the graphs represented data from one mouse. Data were expressed as the mean ± SEM or SD, and *p*≤0.05 was considered significant.

## 5. Conclusions

Based on the present study, we identified that Kvβ2 deletion preserves isoproterenol-induced stress in the heart. This study demonstrated that, while the wildtype mice showed a significant decrease in cardiac function after 14 days of isoproterenol infusion, the Kvβ2 mice demonstrated a preserved function from the isoproterenol-induced responses, as observed by the ejection fraction, ECG, and at the action potential changes. This study therefore provided the basis for Kvβ2 coupling to cardiac excitability and the modulation of contraction, the responses of which are modulated under stress conditions. At the genetic level, we identified key genes that are modulated in Kvβ2 KO mice, which include Bmal1, Clock, and Per3, along with SLC41a3 (solute carrier transporter for magnesium ion). Overall, the decreased expression of SLC41a3 functionally altered the Mg^2+^ responses in Kvβ2 KO hearts, providing the new insight that a lack of Mg^2+^ transporter likely led to an attenuated response to isoproterenol-induced stress in vivo.

## Data Availability

The data presented in this study are available in figure form.
